# Changing national health policies for introduction, uptake and scale-up of self-care interventions for sexual and reproductive health and rights in the Eastern Mediterranean Region

**DOI:** 10.1186/s12961-021-00705-1

**Published:** 2021-04-21

**Authors:** Manjulaa Narasimhan, Briana Lucido, Lale Say, Karima Gholbzouri, Maha El-Adawy, Ahmed Al Mandhari

**Affiliations:** 1 Department of Sexual and Reproductive Health and Research, UNDP-UNFPA-UNICEF-WHO-World Bank Special Programme of Research, Development and Research Training in Human Reproduction (HRP), World Health Organization, Geneva, Switzerland; 2 WHO Regional Office for the Eastern Mediterranean, Cairo, Egypt

“Health for all, by all so that everyone in the Eastern Mediterranean Region can enjoy a better quality of life.” Dr. Al Mandhari, Regional Director, WHO Regional Office for the Eastern Mediterranean

WHO defines self-care interventions as a variety of tools which can be accessed and used by individuals with or without the direct supervision of a health worker. Advances in evidence-based health information, medicines, diagnostics, products and technologies have increased the potential for self-care interventions to increase health coverage for promotive, protective, preventive, curative, rehabilitative and palliative needs of individuals, families and communities. The increasing demand for sexual and reproductive health (SRH) services in the Eastern Mediterranean Region (EMR) and the importance of ensuring universal health coverage (UHC) including coverage, quality and financial protection for SRH also highlights the need for engagement of WHO Member States in the EMR.

The 2019 WHO consolidated guideline on self-care interventions for health (“Guideline”) contains evidence-based recommendations and good practice statements on Sexual and reproductive health and rights (SRHR) [1]. The conceptual framework on self-care interventions for health that informed this Guideline takes a people-centered approach for adopting, implementing and scaling up self-care interventions, and places emphasis on improving people’s health and well-being. The framework is closely linked to the 17 interlinked Sustainable Development Goals and acknowledges that progress in health will require commitment to human rights, gender equality, equity and accountability through two complementary pathways of change to achieve the right to health for all. This includes (1) increasing the autonomy and agency of individuals to make informed health decisions regarding people’s health and well-being; and (2) transforming health systems to create safe and supportive enabling environments that promote and support the use of self-care interventions [2].

Successful introduction and uptake of self-care interventions at the country level will depend on an interplay of factors linking these two pathways, but also on elements that lie beyond the control of individuals and the traditional scope of the health sector. Translating these complex, multilevel and critical changes into effective policy measures requires high levels of commitment and engagement from diverse and relevant partners. Experiences from regions and countries are also needed to make clear what public goods are available to policy-makers, programme managers, health workers and laypersons for harnessing the potential contribution of self-care interventions.

The work undertaken by the WHO Regional Office for the Eastern Mediterranean (EMRO) is an example of how this process can be approached. In 2019, WHO EMRO held several regional- and national-level discussions to disseminate the Guideline and consultative meetings and workshops on integrating self-care interventions for SRH in national health policies, programmes and practices in the EMR. Access to health is undermined by critical shortages of human and financial resources as well as lack of security of SRH essential medicine and commodities in many countries of the region, in addition to tangible physical and logistical barriers and the disruption and insecurity caused by humanitarian emergencies [3]. Following the emergence of the coronavirus disease 2019 (COVID-19) pandemic, EMRO held a regional virtual consultation in April 2020 for rapid implementation of the Guideline to respond to the SRH needs during and beyond the COVID-19 pandemic [4]. Some of the rich and varied experiences in the introduction and scale-up of self-care interventions for SRHR in the EMR have been captured in the papers in this special supplement. The papers in this supplement, individually and collectively, contribute to strengthening the evidence base, as there are gaps in research and in the literature on the highlighted themes. Accelerating progress towards UHC will require continuing to build on successful policies and programmes, such as those highlighted in the papers, as these serve as examples of the essential roles that individuals, families and communities play in promoting and protecting health and well-being, as co-developers of health and social services, and as self-carers and caregivers. With regard to self care, Fig. 1 shows the countries in this region from which data were obtained that informed the respective papers.

**Fig. 1:**
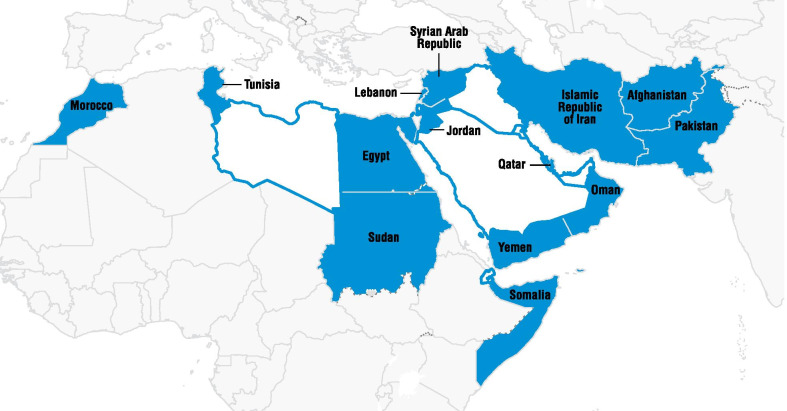
Diversity of settings and national contexts from countries in EMRO that informed the papers in this supplement

Several themes appear across the papers and outline three priority steps for a dynamic approach to national-level policy changes that can ultimately accelerate strengthening and advancing SRHR (Fig. 2). While advocating in EMR for implementing innovative interventions for SRH, considering health system resources, and focusing on vulnerable populations during the COVID-19 pandemic, special attention was given to documenting best practices to showcase countries’ efforts and their contribution in promoting self-care interventions in the EMR.

**Fig. 2:**
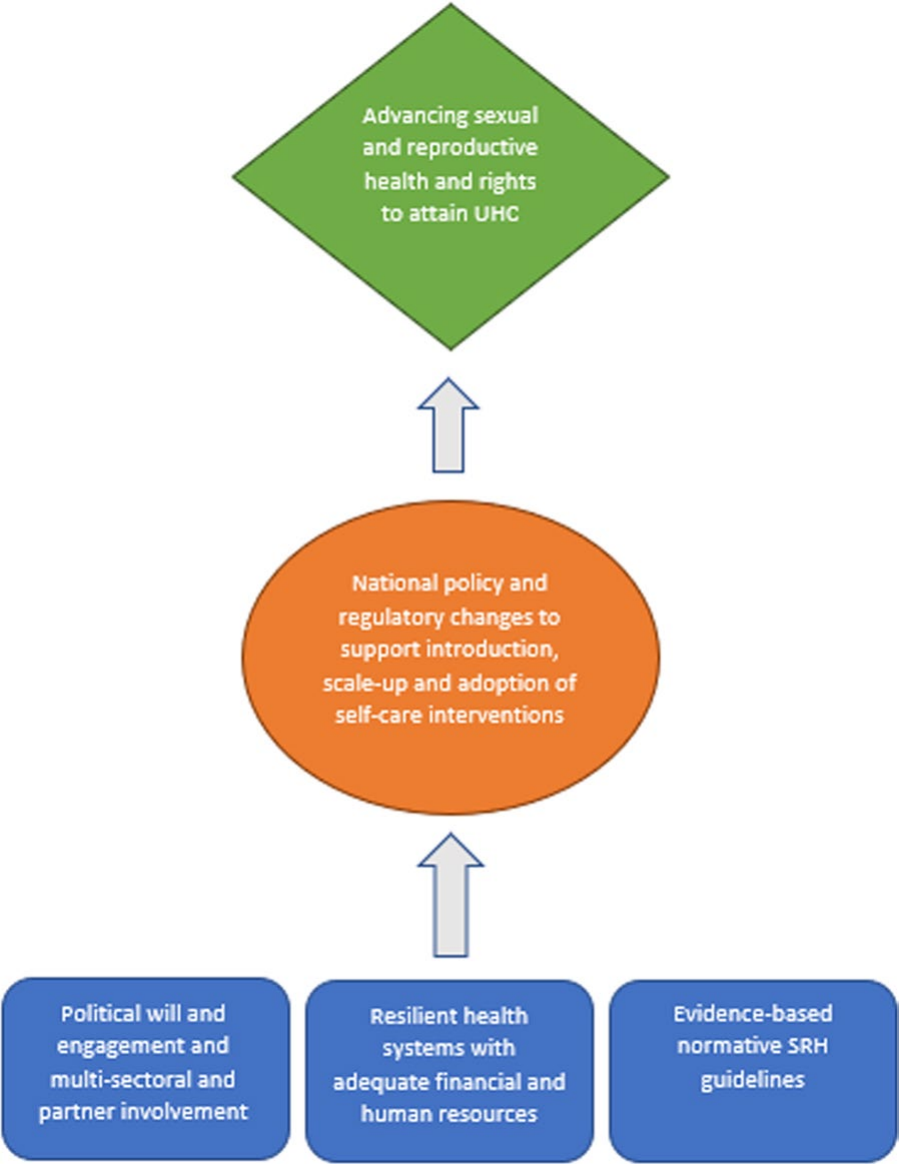
A dynamic approach to policy change at the national level

Firstly, political will and leadership and multi-stakeholder partnerships are required. One example of how catalyzing the political will to act can lead to effective, positive change is shown in the key role played by parliamentarians (Mohamed et al.) to promote innovative self-care interventions through their legislative, budget allocation, oversight and advocacy roles. Public sector ownership and patronage (Uzma et al.) in advocating for better national policies has also been shown to be effective in enabling introduction of self-care interventions.

A second step towards effectively enabling self-care interventions is protecting people’s human rights irrespective of their socioeconomic background, gender, sexual orientation or age in ensuring quality of care. The work of community pharmacists (El Bizri et al.) in increasing coverage of self-care interventions through counselling and over-the-counter access to essential medicines demonstrates how accessing services in pharmacies and outside of traditional health facilities can expand the reach of health services to even the most vulnerable populations. Moreover, even during the pandemic, rights-based approaches to healthcare delivery can improve health outcomes of men who have sex with men (Maatouk et al.), and point to the need to further support policy changes in task-shifting. From a public health perspective, it is advantageous for licensed products to be available over the counter, and the readiness of national regulatory guidelines could lead to increased access, availability and usage of essential self-care interventions (Gülmezoglu et al.). This in turn contributes to ensuring a rights-based foundation for the introduction of self-care interventions.

Thirdly, the papers highlight the importance of evidence-based normative guidance, because they provide trustworthy, reliable sources to inform health decision-making for policy-makers, health workers and laypersons. Results from the regional component (Logie et al.) of a global cross-sectional values and preferences survey give a glimpse of the knowledge and priorities of laypersons and health workers around access, use and uptake of self-care interventions for SRH. Policy-makers can consider the participant narratives regarding the need to consider both the heterogeneity of self-care interventions and the needs and lived experiences of diverse populations to ensure that the policies reflect the priorities and needs of people [5].

The papers included in this supplement provide concrete examples of how policy action leading to implementation of self-care interventions in the EMR can lead to tangible, positive shifts in healthcare delivery. This starts with a constructive policy debate to understand how self-care interventions fit in the context of health, community and policy systems [6]. Policy-makers need sound evidence about what concrete actions people undertake to address health issues, including in their role as caregivers. For instance, principles of gender equality, right to health and equity in access are critical for guiding policy-making decisions and have served in developing services that meet the needs of refugees and key populations with HIV self-testing (Maatouk et al.). It is important for policy-makers to understand what capacities people have for use and uptake of self-care interventions, what resources they can access, and what this means for their understanding of health and of their relationship with health workers and the health system in order to make informed decisions and changes to address health system challenges and capitalize on the roles played by self-care interventions.

Evidence on the patterns of use and uptake of health services and on the effectiveness of specific self-care interventions is not enough to develop effective policies. It must come with better evidence on what strategies work to enhance the capacities of individuals and communities to access quality self-care interventions. A structure to support further research that includes stakeholder values and identification of core gender, equity and human rights standards and principles can strengthen the evidence base [7]. Strong partnerships, collaborative approaches and political will become increasingly important alongside evidence-based instruments and tools to assess the starting point and map the desired directions for policy change.

Several of the papers in this supplement highlight the importance of training health workers to promote and counsel on the use of self-care interventions (El Bizri et al.; Uzma et al.). This will involve training and development of competencies that go beyond biomedical approaches or a disease focus to a more empathetic and compassionate mindset which considers the person at the centre of care and leverages and understands the need for demonstrable supportive attitudes, adequate knowledge and skills; and codes of conducts and specific trainings to eliminate the current power dynamics between health workers and clients. People in different settings and circumstances have varying abilities to access health services, and the ability to promote self-care interventions in populations who may be stigmatized or marginalized and/or lack trust in existing health systems, and to support them as needed with quality care, has the potential to improve health and equitable distribution of healthcare.

Another area of importance is facilitating the linkages between digital and self-care interventions. Although digital health technologies are growing rapidly, access is likely to be unequal due to the digital divide. Closing this gap will require action from policy-makers to ensure sustainable, safe and ethical use of technology in the health sector. Evidence-based, high-quality self-care interventions can be delivered via digital technologies and can offer effective alternatives to some face-to-face interactions with providers. During the COVID-19 pandemic, for instance, such innovative approaches using digital platforms allow health systems to better maintain the delivery of essential SRH services [8].

The experience provided in the papers from this supplement can help other countries accelerate progress on self-care interventions in SRHR. Rapid introduction and scale-up of self-care interventions can be a game-changer for accelerating positive health impact through sustainable action at the local level that is driven by the health needs of people. The examples highlighted from the EMR show how individuals can be are enabled to play an active role in improving their own health (Maatouk et al.), engage successfully with community action for health (El Bizri et al.), and push governments to meet their responsibilities in addressing health and health equity (Mohamed et al.; Uzma et al.). For policy-makers, self-care interventions are important for responding to people’s priorities, needs and rights with regard to health. Ensuring availability of quality, regulated self-care interventions is part of the duty of policy-makers to protect their constituencies against harmful or exploitative practices. Despite the diversity of the countries in the EMR, the opportunities and challenges in the regional examples show the need for quality data (Gülmezoglu et al.; Logie et al.) to inform how the public health community and policy-makers could not just improve health outcomes, but also transform health systems through self-care interventions. Self-care interventions for SRH is crucial component of health systems and their availability is a pre-requisite to ensure SRH service delivery responds to people’s needs and rights to reach UHC.

## Data Availability

Not applicable.

## Abbreviations

COVID-19: Coronavirus disease 2019
EMR: Eastern Mediterranean Region
EMRO: Regional Office for the Eastern Mediterranean
SRH: Sexual and reproductive health
UHC: Universal health coverage
WHO: World Health Organization
